# RE-AIMing COVID-19 online learning for medical students: a massive open online course evaluation

**DOI:** 10.1186/s12909-021-02751-3

**Published:** 2021-05-27

**Authors:** Yusuf Yilmaz, Ozlem Sarikaya, Yesim Senol, Zeynep Baykan, Ozan Karaca, Nilufer Demiral Yilmaz, Levent Altintas, Arif Onan, İskender Sayek

**Affiliations:** 1 McMaster Education Research, Innovation, and Theory (MERIT), and Office of Continuing Professional Development Faculty of Health Sciences, Department of Medicine, McMaster University, 100 Main Street West, 5th Floor, Room 5003, Hamilton, Ontario L8N 3Z5 Canada; 2 Department of Medical Education, Faculty of Medicine, Ege University, Izmir, Turkey; 3 Department of Medical Education, School of Medicine, Marmara University, Istanbul, Turkey; 4 Department of Medical Education, Faculty of Medicine, Akdeniz University, Antalya, Turkey; 5 Department of Medical Education, Faculty of Medicine, Erciyes University, Kayseri, Turkey; 6 Department of Medical Education, Faculty of Medicine, Acibadem University, Istanbul, Turkey; 7 Association for Evaluation and Accreditation of Medical Education Programs, İzmir, Turkey

**Keywords:** COVID-19, Massive open online course, Low- and middle-income countries, Online learning, Medical student

## Abstract

**Background:**

Clinical training during the COVID-19 pandemic is high risk for medical students. Medical schools in low- and middle-income countries (LMIC) have limited capacity to develop resources in the face of rapidly developing health emergencies. Here, a free Massive Open Online Course (MOOC) was developed as a COVID-19 resource for medical students working in these settings, and its effectiveness was evaluated.

**Methods:**

The RE-AIM (reach, effectiveness, adoption, implementation, and maintenance) framework was utilized to evaluate the effectiveness of MOOC in teaching medical students about COVID-19. The data sources included the student registration forms, metrics quantifying their interactions within the modules, students’ course feedback, and free-text responses. The data were collected from the Moodle learning management system and Google analytics from May 9 to September 15, 2020. The research team analyzed the quantitative data descriptively and the qualitative data thematically.

**Results:**

Among the 16,237 unique visitors who accessed the course, only 6031 medical students from 71 medical schools registered, and about 4993 (83% of registrants) completed the course, indicating high levels of satisfaction (M = 8.17, SD = 1.49) on a 10-point scale. The mean scores of each assessment modules were > 90%. The free-text responses from 987 unique students revealed a total of 17 themes (e.g., knowing the general information on COVID-19, process management of the pandemic in public health, online platform use, and instructional design) across the elements of the RE-AIM framework. Mainly, the students characterized the MOOC as well-organized and effective.

**Conclusions:**

Medical students learned about COVID-19 using a self-paced and unmonitored MOOC. MOOCs could play a vital role in the dissemination of accurate information to medical students in LMIC in future public health emergencies. The students were interested in using similar MOOCs in the future.

**Supplementary Information:**

The online version contains supplementary material available at 10.1186/s12909-021-02751-3.

## Introduction

The Coronavirus Disease 2019 (COVID-19) pandemic has affected the medical education in many different ways, as it has impacted all areas of life [1–4]. The uncertainty and chaos in medical schools unprepared in this pandemic, unequal opportunities in accessing educational materials, inadequate course content, and delays in clinical training are the main problems [5, 6]. The medical schools in low- and middle-income countries (LMIC) have limited capacity to develop resources in the face of rapidly developing health emergencies. In addition, the uncertainty about the roles of medical students during the pandemic has made the student participation in patient service a controversial issue [3]. This has led to the adoption of different approaches among the institutions. Some medical schools banned all patient interactions, while the others recruited students for hospital-based roles and had early graduations, so they could serve as frontline clinicians [7–9].

Considering the highly contagious nature of COVID-19, the face-to-face interactions in large group environments (e.g., lecture and tutoring sessions) have a high potential in spreading the disease [2]. The clerkship phase of medical education, which requires active presence in the clinics, is considered as high risk for students in this context. The Higher Educational Council in Turkey recommended all the medical schools to continue their education online. The Association of American Medical Colleges (AAMC) recommended that all student clinical rotations should be discontinued during this period to ensure both the students’ and patients’ safety. They also emphasized that the medical schools take the opportunity to develop COVID-19 training programs to keep all the students updated important guidance for medical students on clinical rotations during COVID-19 outbreak [10].

The disruption created by COVID-19 has allowed the medical educators take advantage of the technology for medical education. While online lectures have been widely used in medical education [2], the COVID-19 pandemic required prompt education and preparation for a wider audience. In this study, a COVID-19 massive open online course (MOOC) and its effectiveness were evaluated.

## Methods

**Fig. 1:**
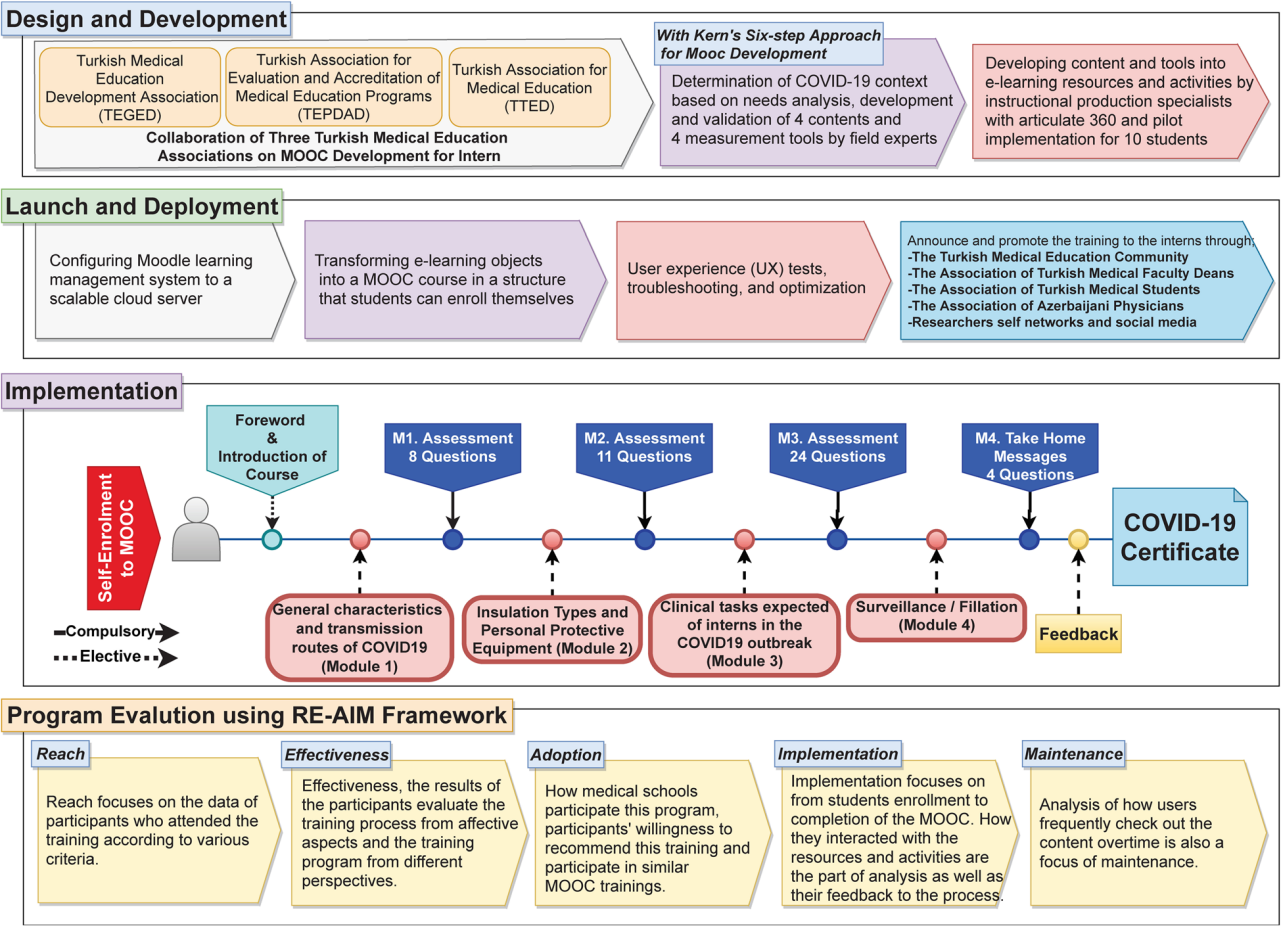
Study design, development, implementation, and evaluation

This study evaluated the RE-AIM (reach, effectiveness, adoption, implementation, and maintenance) framework of a MOOC prepared in an emergency state during the COVID-19 pandemic [11–13]. The RE-AIM framework is an evaluation methodology that has been developing and increasing in popularity for more than 20 years. The framework focuses on both the individual and setting or contextual levels and incorporates the quantitative and qualitative data at all levels [14–17]. It encompasses an overall program evaluation for sustainable and generalizable initiatives and has been used for online learning program evaluation [18, 19]. This framework was used to evaluate our MOOC across all the five elements. Reach is the numbers, demographics, and representativeness of the population. This element was used in terms of how many students accessed, enrolled, and completed the MOOC, along with their current perceived knowledge level as described in the qualitative analysis. The effectiveness element focuses on the efficacy, impact of the outcomes, and negative effects of an intervention on an individual level We utilized the effectiveness element to examine the students’ learning and satisfaction with MOOC. The feedback form and quiz results were used to understand if the MOOC was effective and how it contributed to the students’ learning. The third element of the RE-AIM framework is adoption, which describes the numbers and proportions of the institutions that participated in the intervention at a setting level. Adoption defines how people (e.g., staff, faculty, and student) can be involved in the intervention on an individual level. This study used this element to understand how medical schools participated in the MOOC and recommended it to their students as well. We also examined the students’ uptake, feelings, and willingness to be involved in the program through a feedback form. Implementation defines the key functions and/or components of an intervention, including its consistency, delivery, time, and cost. In this study, this component was analyzed to show how the MOOC was designed, developed, and implemented at a large scale, along with the modules and quizzes completion statistics. Lastly, maintenance at a setting level indicates that an intervention becomes institutionalized or embedded in the routine practices in a sustainable way and the long-term effects of an intervention after the completion of a program on an individual level. In this study, we focused on the individual level maintenance, in which the content was updated throughout the pandemic and how the students visited the MOOC at different times was analyzed. Figure 1 summarizes the overall program evaluation process, from its development to the evaluation phase of the course.

### MOOC development and implementation

While there were many uncertainties about COVID-19, researchers faced the challenge to design and develop a MOOC program, which introduces and teaches the current scientific disease management approach for COVID-19. It was crucial to rapidly develop a training program for medical interns during the pandemic. In these exceptional cases, we combined the instructional design principles with the curriculum development processes in a pragmatic approach because some outputs of the instructional design and curriculum development models serve as the inputs for the other. Thus, we combined the “analysis, design, development, implementation, and evaluation” (ADDIE) instructional design model with Kern’s six-step curriculum development approach, with a systematic approach to make MOOC available for large scale participation [20–22]. From problem identification and needs assessment to the last step of evaluation and feedback, four modules and module assessment tests were developed: general characteristics and transmission routes of COVID-19 (Module 1), insulation types and personal protective equipment (Module 2), clinical tasks expected of interns in the COVID-19 pandemic (Module 3), and surveillance/filiation (Module 4) on the Articulate Rise platform.

Using these modules, an instructional design was developed on a Moodle learning management system (LMS), and 10 medical students were included in a pilot test for the overall process. The MOOC was made available to the public online at https://online.tepdad.org.tr/course/view.php?id=14, but only the registered users were able to access the assessment modules. During the implementation phase, the students who enrolled in the MOOC themselves first encountered a preface and an information video broadcasted by our senior author (IS) – a well-known respective medical educator in Turkey. Then, the modules and assessment activities (i.e., quizzes) were accessed. Each assessment module contained various questions to examine its learning objectives. The students were required to wait for a 5-min grace period before starting a new attempt on the quiz. Finally, they were awarded with an online MOOC completion certificate upon successful completion based on the 80% passing grade on all assessment activities. In addition, a feedback form was filled out to evaluate the MOOC in terms of its technical and educational content.

### Participants

The target group of the MOOC included the medical interns (6th year undergraduate medical students) from Turkey, but the registration was free and open to everyone. Therefore, the medical students from other year levels as well as the other users (e.g., residents, faculty) were able to enroll in MOOC. However, our participants were medical students whose data were included in this study, and those of the others were excluded.

Our main method of participant requirement was to formally invite all medical schools in Turkey through TEPDAD (Association for Evaluation and Accreditation of Medical Education Programs-Turkey). We used several methods of participant requirement including the medical student communication groups (e.g., Facebook groups, WhatsApp groups, and other social media), Turkish medical education student network, Turkish medical education community, Deans’ Council of Medical Schools, and the research team members’ personal communication network and social media. We also contacted the Association of Azerbaijani Physicians to announce the MOOC through their networks.

### Data collection tools

LMS was used to collect various data at the different levels of MOOC, including the user registration form, module assessment results, and students’ course feedback (evaluation) form (Table 2), which were the main data sources within this program evaluation study (Supplement 1). We also gathered the Google analytic data to examine the MOOC reach campaign. The data collection window was from May 9 to September 15, 2020.

### Analysis

Google analytics were reported descriptively. The user registration data, which consisted of several demographics (e.g., gender and grade), were used to compare the cohorts for module assessments and course completion. Descriptive statistics was also utilized to analyze the students’ demographics, online system logs, course feedback, and module and assessment completion statuses. The analysis was reported under the RE-AIM framework elements.

The students’ course feedback form included free-text questions, which consisted of rich data for the MOOC. Four open-ended questions that made up the students’ reflections in the course evaluation form were analyzed qualitatively. At this stage, the qualitative analysis team consisting of three researchers (OS, ZB, and YS) decided to use the thematic content analysis method. Initially, each open-ended question was analyzed and coded by one of the researchers. The second researcher independently re-coded the same data set. The third researcher the main and sub-themes by examining the codes created by the first two analysts. To support the themes, exemplary quotes from the students’ responses and their qualitative username, which combines the response identifier and gender code, were selected. These themes were presented in the context of RE-AIM framework. The results of the themes with the quotations were also presented under the framework elements (Table 1).

**Table 1:** Thematic analysis of the students’ course feedback

Framework Element	Theme	Example Quote
**Reach**	Knowing the general information on COVID-19	*I had a lot of information regarding each topic provided within this course. In this course, I was able to learn more details and specifics on approaches to treatment. (292 W)*
	Various sources of information gathering	*I have regularly followed the guidelines shared by the Ministry of Health and WHO compared to the course content. That’s why I felt like I was in control of the issues in general. (463 M)*
	Broadcast and media effect on COVID-19 knowledge	*I had some information that I read from the news and heard from the media. I was also looking up the details but this course provided me a lot of information that I was curious about. (1628 W)*
**Effectiveness**	Basic medical knowledge on COVID-19	*I have learned the contamination sources, and that mothers can breastfeed their babies with a mask by following the hygiene rules. (756 W)*
	Process management of the pandemic in public health	*I learned what surveillance and radiological methods are and why they are done. (125 W)*
	Diagnosis and treatment algorithms to approach patients with possible COVID-19 and/or risk groups	*I had knowledge about how to approach patients, but I did not have a complete command of the algorithms. I have learned better now about contamination times and locations. (600 W)*
	Diagnosis and treatment of patients with possible COVID-19	*Since the universities were closed with the start of the pandemic, I was able to continue to learn with my own efforts. I only had some basic information that I had learned from microbiology / infection internships. But now, I have learned comprehensive information on many topics such as the approach to the patient and personal protection methods. (371 W)*
	Health of healthcare workers	*I have learned better how I can better protect myself against COVID-19, and prevent transmission to colleagues and patients. At the end of the course I felt more confident and stress free. (318 W)*
**Adaption**	Online teaching during the quarantine times	*I think it is definitely the best investment I have made for myself during the quarantine, in the process that we are excited to be an intern in these difficult days and we are very excited about being a doctor. (907 W)*
	Course recommendation with perceived benefits	*I really enjoyed this course - it was very well-organized and interactive. I learned many new things regarding COVID-19, especially information that is relevant to the clinic/ hospital setting. Highly recommended to anyone working in the healthcare field. (2020 W)*
**Implementation**	Use of online platform and Instructional design	*The course was overall well above what I expected. The content design was very well-organized. I like that step-by-step, different interactions at each step, short question-answer when necessary, and supported with videos. It kept my interest and gave me directions at each step to think about more on the topics. It has been a useful program by keeping the attention solid, without just going through it. (458 W)*
	Standardization on knowledge of the COVID-19	*Supporting the intern training in this way provided equal opportunity to everyone to learn standardized and centrally distributed information which was provided from the experts in the field. I hope there will be other similar courses in the future. (219 M)*
	Content improvement	*I think a more effective course can be done with more visual materials and live lectures. A platform can be created where we can listen to experts and general practitioners who have been in the field and experienced the approach to the pandemic. (1687 W)*
**Maintenance**	Updating current practices and algorithms	*I would love to learn current treatment procedures for COVID-19. Especially, we hear from our peers that a different guideline has started to be used from the world. I would like to learn in detail the approach and diagnosis stages of the world as well as our country’s approach to the COVID-19 treatment. (1339 M)*
	Following up on the unknown topics during the pandemic	*I would like to know about new drug and vaccine development studies for COVID 19, approaches to the patients with COVID-19, and epidemiological data from different countries. (1096 M)*
	Changes in patient management	*I would prefer more concrete examples on the topics. For example, like a case report, the complete findings of a patient, their examinations and results (*e.g.*, lung X-ray, CT image, blood work, blood gas), applied treatments, and clinical course could be more effective for our learning. (1784 W)*
	Stress management	*I would like to know more about stress management. In the current period, I think that the most tiring situation for me will be psychological wear rather than the physical work. The most important things will be the attitudes of patients and their relatives, our communication style with our colleagues and the level of working conditions. I would like to get personal views from different people who are actively working on how they handle these situations. (501 W)*

### Ethics

The Hamilton Integrated Research Ethics Board granted an ethical exemption because this was a program evaluation study. All methods were carried out in accordance with the relevant guidelines and regulations. An informed consent was obtained from the participants who filled out the feedback form.

## Results

### Reach

From the announcement of the course on May 9, 2020 to September 15, 2020, Google analytics showed that 16,237 unique visitors accessed the MOOC. Of the visitors, 37% (*n* = 6068) enrolled in the MOOC. After removing the non-medical student users and fake accounts, we included 6031 medical students in our analysis. While most of the registered users were from Turkey, there were enrollment from 18 different countries. Of the students, 54% were female, 77% were interns, and 14% were 5th year medical students. The mean age of the students was 24.18 (SD = 1.72). The students represented a total of 71 universities, and the majority of the universities were from Turkey.

In total, 2245 students filled out the feedback form with a response rate of 37%. The first section of the feedback form included Likert items, while the second section had four free-text questions which were not mandatory to complete to submit the form. In total, 987 unique students (16%) responded to the free-text questions. The number of students’ responses for each question are as follows: 760 (13%) responses for “What did you already know about the content of this course?”, 753 (12%) responses for “What are the main topics you have just learned with this course?”, 724 (12%) responses for “Which subjects would you like to have more detailed information about?”, and 653 (11%) responses for “Want to add?”. During the analysis of the responses, we excluded some responses as they do not contain any useful information (e.g., ‘thank you’ and ‘No’). Table 1 presents the qualitative analysis of the free-text responses based on the framework elements from the course feedback forms.

Qualitative analysis related to the reach element showed three themes, which are ‘knowing the general information on COVID-19’, ‘various sources of information gathering’, and ‘broadcast and media effect on COVID-19 knowledge’. The results showed that while some students (*n* = 247, 4%) reported that they did not have any information about the COVID-19, 9% of the students (*n* = 572) already had the information about the microbiological properties, transmission routes, epidemiological data, diagnosis, treatment and prevention methods, epidemic management, and use of personal protective equipment (PPE). One student commented on their prior knowledge as “*I knew the importance of wearing a mask and washing hands, filiation as effective in finding the source of contamination, most of the patients had mild or asymptomatic disease, the incubation time of the virus, and the rapid antigen test (1696W)*”. Of the students, 1% (*n* = 90) stated that they have been following multiple sources of information (e.g., Ministry of Health and World Health Organization) before the course started; however, they also added that their knowledge on the topic was similar to a lay person before they took the course, not enough as a prospective physician. The knowledge gap showed that the MOOC’s reach was the medical students who have known the pandemic as little as the public does.

### Effectiveness

Of the students, 83% (*n* = 4993) completed the MOOC successfully and received their electronic certificates. There was no significant association between gender and course completion (χ^2^(1) = 2.27, *p* = .132).

Table 2 shows the students’ course feedback results. The overall satisfaction was high (M = 8.17, SD = 1.49) based on a 10-point scale. The first part of the questionnaire also asked the students how they felt about the course. The results showed that the students benefited (M = 4.48, SD = .70) and learned (M = 4.42, SD = .69) from the course and focused (M = 4.06, SD = .77) on it based on a five-point scale. Although there were no interactions between the users on the online platform, the students did not feel alone (M = 1.69, SD = 1.03) while using the MOOC.

**Table 2:** Students’ course feedback questionnaire results

Questionnaire Item (***n*** = 2245)	Mean	Standard Deviation
**At the end of this course, ... (1-Never … 5-Always)**		
1. I benefited.	4.48	0.70
2. I learned.	4.42	0.69
3. I got focused.	4.06	0.77
4. I enjoyed	3.74	0.95
5. I struggled.	2.44	0.94
6. I got bored.	2.28	0.96
7. I got tired.	2.15	1.03
8. I felt alone.	1.69	1.03
**Evaluate the course in different aspects with the following items (1-Strongly Disagree … 5-Strongly Agree).**		
9. I recommend this course to all physician candidates.	4.32	0.77
10. I would like to receive similar courses designed in this way.	4.13	0.86
11. The course was well-organized.	4.12	0.86
12. The length of the modules was appropriate.	4.10	0.83
13. I can use what I learned in the clinic.	4.10	0.72
14. The modules were prepared in accordance with the learning objectives.	4.09	0.90
15. Course content was sufficient.	4.05	0.86
16. End of module questions were sufficient to evaluate my learning.	3.79	1.07
17. At the end of this training, I feel ready to work in the clinic.	3.58	0.92
18. I would like to share on the system with my friends who attend the course.	3.43	1.11
19. I would prefer a live lesson (with the lecturer’s synchronous narration) in the course.	3.16	1.19
20. I had technical problems while using the system.	2.01	1.25
21. I had internet access problems while completing this course.	1.78	1.14
*22. Overall course satisfaction (Between 1 and 10-point scale)*	8.17	1.49

The students self-reported on a free-text question that asked about what they had learned in the course. The thematic analysis of 753 responses on the new knowledge piece resulted in five themes (Table 1): ‘basic knowledge of COVID-19’, ‘process management of the pandemic in public health’, ‘diagnosis and treatment algorithms to approach patients with possible COVID-19 and/or risk groups’, ‘diagnosis and treatment of patients with possible COVID-19’, and ‘health of healthcare workers’.

The first theme was the basic medical knowledge on COVID-19. The students explained their learning points on the microbiological characteristics of COVID-19, the clinical picture of the disease, the transmission routes of the virus, and the protective measures for these transmission routes. One student listed their key takeaways as “*What the COVID-19 is, its difference from other respiratory infections, transmission ways, and protection ways (899M).*”

The second theme of the process management of the pandemic in public health was about the community-based approach used in the COVID-19 outbreak management and primary health care. A significant number of students stated that they learned about the surveillance / filiation methods, especially in the new epidemic management in this course. The sub-themes included the community-based approach in outbreak management, search for sources of contamination, isolation of patients and protection of their relatives, types of surveillance to be selected according to the size of epidemic and health problems, importance of primary healthcare services in epidemic management and the duties of the healthcare team, preventive health services, and health education issues. A student summarized the key takeaways for the course as “[I have learned] *how I can approach the COVID-19 suspicious / definite case at the periphery... How the radiation study can be done, how the cases are reported … (169W)*”.

The third theme related to the newly learned subjects of the students participating in the course was about the diagnosis-treatment algorithms developed and updated to approach the patients with suspected COVID-19, its definitive diagnosis, and the Ministry of Health guidelines. Approaching high risk groups, such as pregnant, lactating mothers and transplant patients, was among the topics that attracted the students’ attention. One student emphasized the value of the course by asserting that “*I learned … approaches about pregnant women and immunosuppressive patients thanks to this course (1901W)*”.

The fourth theme was the information on the diagnosis and treatment approaches of patients with suspected COVID-19. One of the topics that the students emphasized was that they used the visual course materials about the oropharyngeal swab sampling for diagnosis. In addition, home treatment, isolation, and follow-up of patients diagnosed with COVID-19 and the drug interactions in the treatment process of patients with poor prognosis were among the newly learned topics. A comment from a student summarized the importance and effectiveness of the MOOC as “*I have learned in detail the clinical symptoms, course, diagnosis and the latest state of the treatment of COVID-19 in the world. My anxiety about what to do in the internship decreased. I feel more confident because I know what to do (1254W)*”.

Lastly, the fifth theme was on the health of healthcare professionals and the ways of protecting them. The students who participated in the program emphasized that they benefited from the video and checklists for PPE usage and standard PPE wearing order. One student commented the outcomes of the course for their learning as “*The order of wearing and taking off personal protective equipment, how to apply hand disinfectant to our hands, whether the medical mask is sufficient when entering the patient’s room (1310W)”*. The students reported that they learned about these five themes during the course.

### Adoption

The students from 71 different schools registered in the MOOC. After TEPDAD sent a formal invitation to the medical schools to participate in the course by announcing it to their students, our data showed mass attendance from several universities. Some of them made student certifications mandatory through the online platform. The number of students within the same university ranged from 1 to 723, with a mean of 83 (*SD* = 149). There were 24 universities which sent at least 50 students or more with a total of 5699 students.

The student course evaluation also highlighted some aspects for adoption (Table 2). The highest ranked item was the students’ recommendation of the course to their peers (M = 4.32, SD = 0.77). Moreover, the students expected similar MOOCs in the future (M = 4.13, SD = 0.86) and found that the course was well-organized (M = 4.12, SD = 0.86). On the other hand, the students had less internet access (M = 1.78, SD = 1.14) and technical (M = 2.01, SD = 1.25) problems during the course.

Qualitative analysis revealed two themes (Table 1) for adaption, which were in line with the quantitative results. The students’ feedback highlighted some aspects of the positive contribution of the course (Table 2). Online teaching during the quarantine was an important way to reach the students and support their self-paced learning and prepare them as prospective physicians. The students perceived the benefits of the course and found it important for their peers to learn and get ready for the COVID-19 pandemic. One student described their experiences as “*definitely the best investment I have made for myself during the quarantine … (907W)*”.

### Implementation

The MOOC was opened to the public, so anyone was able to browse the content without registration. Therefore, the website received a lot of guest users, and browsing without registration also saved us from fake accounts and unnecessary user registration. In order for the users to get certified, user registration was required. From their enrollment to the course, the students completed four modules with an assessment quiz following each module. The questions in the quiz were written according to a blueprint created from the learning objectives of each module. We analyzed the completion of each module and quiz. When students opened a module and saw 80% of the module content while interacting with it (e.g., clicking the next button, scrolling on the content, and answering a knowledge check question), the LMS reported that they completed the module. Similarly, when the students took a quiz and submitted their answers, the LMS reported that the students finished the quiz. The analysis of this completion reports showed that the students completed the modules and quizzes at a different rate. The completion rate of the modules and quizzes were as follows: 62% in Module 1 and 86% in Quiz 1; 65% in Module 2 and 86% in Quiz 2; 67% in Module 3 and 84% in Quiz 3, and 66% in Module 4 and 84% in Quiz 4. Table 3 shows the detailed analysis of the quiz results of the students. The module’s assessment mean scores ranged from 90.14 (SD = 10.68) to 99.47 (SD = 4.09). The students were able to repeat the quizzes as many times as they desired, but there was a 5-min grace period before starting a new attempt if a student failed on the quiz. The students were able to surpass the passing grade of 80% on their first attempts for Quiz 2 (M = 87.56) and 4 (M = 89.00), and they were close to pass on Quiz 1 (M = 74.38) and 3 (M = 78.34). The students did not need many attempts to pass the quizzes as their average attempts ranged from 1.10 to 2.44. The results suggest that the students struggled the most on Quiz 1.

**Table 3:** Quiz results for each module

	Quiz 1	Quiz 2	Quiz 3	Quiz 4
Number of questions in the quiz	8	11	24	4
Number of students took the quiz	5515 (91%)	5241 (87%)	5181 (86%)	5137 (85%)
Number of students did not take the quiz	516 (9%)	790 (13%)	850 (14%)	894 (15%)
Number of students had passing grade	5160 (86%)	5176 (86%)	5070 (84%)	5045 (84%)
**Highest grades (i.e., final grades) quiz results analysis**				
Mean	90.14	93.18	96.15	99.47
Std. Deviation	10.68	8.48	7.86	4.09
Median	87.50	90.91	100.00	100.00
Mode	87.50	100.00	100.00	100.00
**First attempt quiz result analysis**				
Mean	74.38	87.56	78.34	89.00
Std. Deviation	20.48	14.32	17.53	15.97
Median	75.00	90.91	79.17	100.00
Mode	87.50	90.91	100.00	100.00
**Average number of attempts (standard deviation)**	2.44 (2.5)	1.10 (0.65)	1.41 (0.78)	1.44 (1.29)

Three themes (Table 1) emerged for the implementation element: ‘use of online platform and instructional design’, ‘standardization of the knowledge about COVID-19’, and ‘content improvement’. The students expressed that the MOOC was very helpful in reducing their anxieties and uncertainties caused by the issues of abrupt graduation prior to the end of the regular academic year due to the pandemic. One student compared their experience to other online courses about the COVID-19 as “*I had accessed a course similar to this from the WHO website. This course was better prepared and structured compares to the WHO’s course. I am grateful to all faculty who contributed in the course. Thank you very much for working for us without ignoring the future physicians as well as fighting the epidemic (1156M)*”. They also mentioned that the MOOC corrected some of the misinformation and misconceptions that were being broadcasted on the television and social media. The students also indicated that the instructional design of the modules was well-organized, and the visuals made it easier to understand the subject. Moreover, the students expected more contents on the diagnostic radiological imaging and treatment. They also mentioned that some questions in the assessment modules could not be answered without further information. In addition to the asynchronous modules, some students requested synchronous interactive live sessions to learn and discuss the complex cases that they would likely encounter in their future practice.

### Maintenance

**Fig. 2:**
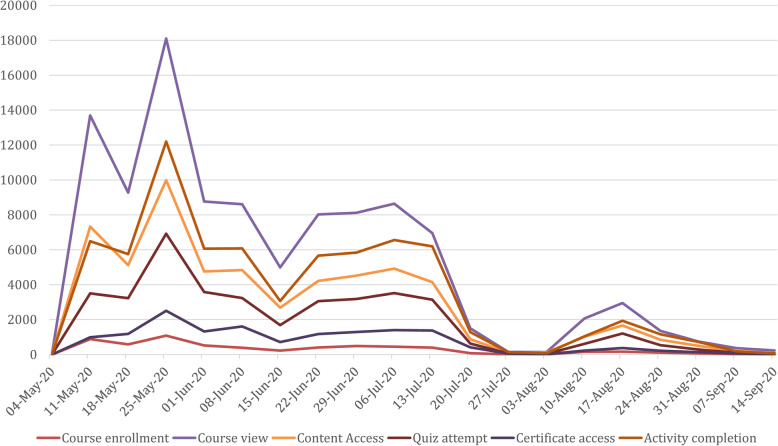
Student course interaction’s frequencies over time by engagement type

As the last step of the RE-AIM framework, maintenance required continuous updates. The research team closely followed the developments in the COVID-19 pandemic and updated the course content. As a result, we included a major update in all four modules during summer. This update helped the students revisit the course contents repeatedly. Figure 2 shows the students’ online activities based on the weekly logs. Updating the course contents clearly showed that the students needed a reliable source of information while the COVID-19 pandemic continues. Therefore, the research team will continue to update the course contents until the pandemic ends. Although it was not included in the analysis, there are currently 7314 registered users on the MOOC as of December 1, 2020.

The maintenance element consisted of four themes: ‘updating the current practices and algorithms’, ‘following up on the unknown topics during the pandemic’, ‘changes in patient management’, and ‘stress management’ (Table 1). The students stated that they wanted to follow the current epidemiological data (e.g., changes in the characteristics of transmission, mutations, and risk groups) while the pandemic evolves and to learn about the changes in the algorithms in approaching patients via case definitions. Some students’ expectations were related to the issues that were not yet answered during the COVID-19 pandemic at the time of MOOC maintenance. The students expected us to update the course contents based on the current practices (e.g., algorithms to approach patients with COVID-19 and epidemiological data on transmission routes and high risk groups). As requested by the students, during the summer 2020, the course content was updated with the current practices. However, some factors associated with the updates were unknown (e.g., vaccine development, treatment protocols, and immune response). The online platform eased to update the course content constantly when the guidelines related to COVID-19 were changed. As reported in the qualitative analysis, the students reported that the updated content helped them access the most up-to-date information while the pandemic unfolds. The students mentioned that they would appreciate the imaging and laboratory findings in the course contents, holistic information on patient management during the pandemic, and stress management for the patients and their relatives. The students also considered the learning experience on stress management as crucial to communicate with the patients and their relatives during the pandemic in the context of professional attitudes and behaviors. One student highlighted the future topics to be considered as “*how I can approach and support patients psychologically, calm them down and convince them to follow the rules of social distancing. (197W)*”.

## Discussion

The rapid and unprecedented spread of the COVID-19 pandemic worldwide has greatly affected public health. A number of measures have been taken for COVID-19, including the ceasing classroom education (i.e., in-person teaching and learning) in medical schools. At the beginning of the COVID-19 pandemic, the medical schools in our country suspended the direct student participation in patient care to overcome the effects of the pandemic. During this period, the interns were sent home in a short period of time with the uncertainties of how they could continue their education. An online training program was developed in collaboration with the three medical education associations in Turkey (TEPDAD, TEGED, and TTED) focusing on the scientific and practical problems of COVID-19 and how exactly the interns would continue their education in the training hospitals. This study included the qualitative and quantitative program evaluation of COVID-19 MOOC using the RE-AIM framework.

Previous studies confirmed that online education can be used to educate health professionals [23–26]. In our study, a MOOC was developed since the medical students’ needs met its purpose, provided the data on how they responded to their perceived benefits, and focused on the course as well as the results of the assessment on learning. In the beginning of the pandemic, preparing the course in Turkish language contributed in preparing the students to protect themselves and their patients as much as allowed by the constraints in their fields. The results highlights that most students self-reported that they benefited from the course, and our assessment results supported this with the students’ grade of ≥90% in the assessment quizzes. On the first attempt, the students scored close to the 80% threshold for Quiz 1 and 3 and passed the threshold on Quiz 2 and 4. With our grading method of highest grade, the students were able to surpass the barrier easily on their second and proceedings attempts. Between the attempts, the students had chance to explore the module contents while waiting for the grace period for the next attempt. This approach allowed them to engage in the content and think about the topic. They highlighted that they learned new information about COVID-19 (e.g., community-based pandemic process, approach to the suspected patient, and diagnosis-treatment algorithms). The highest level of positive evaluation in the students’ feedback was that they benefited, learned, and focused from the course. In the examples of the feedback thematic analysis about the student course, there were some important motivational definitions, such as “*The most important investment I made in myself in these difficult days and it excited me to become a doctor (907W)*”. Most students stated that they would recommend this course to physician candidates. It shows that providing the students with learning activities as outlined in the program development and instructional design principles of the MOOC, such as video, case analysis, and end-of-topic evaluations within the scope of the module subjects, could improve the learning outcomes, motivation, satisfaction, and attitudes of the students [27]. The technology acceptance model emphasized that the ease of use is an important consideration in implementing technological innovation [28]. We made a very basic instructional design with a minimum barrier for students’ access to the system, rather than complicating the system with many conditional activities where a student needs to complete a module and take the next one. Our design approach with Kern’s six-step approach helped us achieve this method to create a flexible method of accessing the content. The students’ feedback on the modules and overall course design increased the motivation, attitudes, and satisfaction by fulfilling the students’ expectations and aimed outcomes. Throughout the MOOC implementation, several technical complaints were received (e.g., not receiving the registration confirmation email), but none of these emails were for how to use the system and completing the contents. Implementing a MOOC should always avoid the technical difficulties on how to use the system and complete a course.

Interns, as the final-year medical students, are clinically important in the healthcare workforce [29, 30] of Turkey. Some certified courses, such as Advanced Cardiac Life Support and Pediatric Advanced Life Support, were delivered online in less than a week [31]. This course, in which a large number of interns attended, contributed to the preparation of interns for the COVID-19 pandemic.

The results confirm that the participation in and completion of the MOOC was higher compared to that of previous studies that showed big dropout rates in other MOOCs [32, 33]. The MOOC topic contributed to this high completion rates. The widespread post-truth information through social media created a questioning of information. On the other hand, our MOOC created and disseminated a very respective association of medical education nationally and internationally. This contributed to this high completion rate and students’ attention. Besides, medical schools which have low resources to train their students encouraged them to complete this course and provide the certificate back to the faculty administration to ensure that their students have the required basic skills. Therefore, providing a national support for a certification process helps in high achievement rates. Moreover, we estimated that there are currently approximately 10,000 student interns based on the medical student enrollment quota in 2013 [34]. The results show that the MOOC reached almost half of the interns in Turkey. Most students who answered the pre-course questionnaire already knew some general information about COVID-19. This result is also in line with an initial study from Turkey conducted in the beginning of the pandemic in April [35] and studies for Bangladeshi and Iranian students [36, 37]. In a study conducted on the students in Mumbai, the percentage of correct answers in the questionnaire was found to be 74.1% [38]. While the survey questions for Iranian and Bangladeshi students were aimed to their knowledge, skills, and attitudes, our results based on self-assessment indicated similar results. The students who attended the MOOC expressed and showed that their knowledge and attitudes to battle this pandemic increased. The fact that the COVID-19 pandemic is a current issue and students follow other resources may be the reason behind their motivation and engagement on the learning materials presented on MOOC.

The students reported that they were using various sources of information regarding COVID-19, especially from WHO, the Ministry of Health in Turkey, and social media. They also emphasized that social media had the information for lay people, rather than medical-level information. While many studies in social media are related to information gathering [39], our students found that social media did not provide useful information for their education, similar to the findings of another study [40]. We acknowledge that these posts in Turkish language may not provide adequate information for the students. Another study reported that the Turkish medical students accessed the COVID-19 information on social media (32.8%), Turkish Ministry of Health website (30.7%), and WHO website (14.5%). Thus, more Turkish contents should be provided to the students on social media. Besides, the WHO website provides an open MOOC in various languages, excluding Turkish [41], and this was the major barrier for our students to be informed about COVID-19. Therefore, this MOOC was created and had a positive impact on the students.

The lowest average student participation and passing grade was for “Surveillance / Filiation (Module 4)”, and the students felt least competent about this topic based on their qualitative evaluation. Improving the functions of health professionals in delivering services and guiding the community in their preparedness are crucial during extraordinary situations from the first response to the recovery phase [42]. A study in 2006 reported that the curricula in 37 Turkish medical schools had limited hours and learning activities on health services in unusual situations [43]. In a study in which medical students reported their basic medical competencies, around 70% of them were confident about their competencies on basic medical practices, and 56% were confident in providing health services in extraordinary situations and providing health education to the community [44]. Similarly, infectious disease reports and case follow-ups among the medical students are limited [45], suggesting that the medical curricula do not have enough focus on these topics. These results are also in line with our findings where medical students reported the lack of information about surveillance and filiation. Therefore, we suggest developing new learning resources for surveillance and filiation.

One of the topics that the students emphasized in the feedback was regarding the use of PPE. In particular, the order of wearing and taking off PPE was mentioned. The interns, who will be a part of the healthcare team during the COVID-19 pandemic, have a high risk of exposure to infection given their limited clinical experience [37, 38, 46, 47]. While the rate of wearing a mask was very low in the initial stages of the pandemic [39], it is noteworthy that the use of masks increased with the prolongation of the COVID-19 pandemic.

In our study, stress management was among the topics that the students emphasized in their feedback about the educational program. They stated that more information should be provided on this topic. The students noted that stress management during the pandemic is a vital issue to focus on, and they also stated that effective communication and teamwork are important competencies in these stressful conditions. The misinformation- and misconception-induced anxiety was strongly discussed in the feedback given by the students who attended the course. The students reported that participating in the MOOC reduced their stress levels and discussed the effect of correct information and effective communication on stress management. Stress management was among the topics that the students participating in the course emphasized about the education program. They stated that more information should be provided on this topic. This topic is also important internationally, since many students have been coping with severe levels of anxiety [48, 49]. Online support can help the students by clearing the unknown and unprecedented issues around their education [50]. Unfortunately, there was no such support mechanism in our study, so we suggest that the researchers should focus on this in future studies.

The analysis of the students’ feedback revealed four themes for continuous maintenance. In addition to the course content updates, the multi-central characteristics of the research team and the supporting bodies of MOOC will create new similar MOOCs for medical students. This may help low-resourced faculties for centralized content, since the upcoming academic year was moved to online classes in many universities. Creating similar MOOCs may decrease the workload and give universities more freedom to enrich their online teaching.

### Limitations

The main limitation of the study was that there was not a human oversight for this MOOC, and the students learning was not reinforced by a course moderator. While we constantly improve the course content by following the rapidly evolving guidelines during the COVID-19 pandemic, we were not able to cover every aspect of it. Moreover, majority of the students who received their certificate did not revisit the updated content. Due to the rapidly changing nature of the pandemic, newer certification process is needed for the updated information. Finally, we have given maximum efforts to provide random quiz questions suitable for the students. The students might have shared the quiz questions outside the system, which might affected the quiz results.

## Conclusions

The governing bodies of a MOOC have a vital role for medical schools that adopt and implement the course at an institutional level and for reaching out to medical students in times of crisis. Centralizing the same content for many medical schools would contribute to standardize the educational outcomes. To conclude, the medical students benefited from the self-paced and unmonitored MOOC on COVID-19, and the feedback of the students showed that there are areas for improvement in the future MOOC.

## Supplementary Information

**Additional file 1.**

## Data Availability

Materials are free and open to everyone at https://online.tepdad.org.tr/course/view.php?id=14 Data is available upon request. Please contact to yilmazy@mcmaster.ca for inquiry.
