# Supplementary material for: RE-AIMing COVID-19 online learning for medical students: a massive open online course evaluation
Source: BMC Med Educ. 2021 May 27;21:303. doi: 10.1186/s12909-021-02751-3 (PMC8154107; doi:10.1186/s12909-021-02751-3)
Supplement: Supplementary file 1 — Additional file 1. [file 12909_2021_2751_MOESM1_ESM.pdf]

Feedback

1 * Gender

- Female
- Male

2 * Your Birth Year

e.g.***1998***

Do not use thousands separators.

3 * your University

4 * Have you ever attended any online training with professional or social content certification?

Yes No

5 * In which range do you see yourself in using internet technologies:

1 I have little knowledge of internet technologies ------- 10 I know and use Internet technologies at a high level?

1 2 3 4 5 6 7 8 9 10

6 * Which or which technologies did you take this course with?

- Mobile (smart) Phone
- Tablet (ipad vs.)
- Laptop
- Desktop hard computer
- Other

7 * Which link did you use?

- Mobile internet (Mobile phone internet)
- Wireless (Wi-Fi) broadband internet
- Other

8 * Did you need financial support (example: extra internet, computer) to participate in this course?

Yes No

9 * At the end of this training ...

	Never	Rarely	Sometimes	Often	Always
I learned.					
I am bored.					
I got tired.					
I enjoyed					
I was struggled.					
I benefited.					
I felt alone.					
I'm focused.					

10 * Evaluate the course in different aspects with the following items.

		I strongly disagree	I do not agree	undecided	I agree	Absolutely I agree
Course content was sufficient.	◉	○	○	○	○	○
The course was well organized.	◉	○	○	○	○	○
The modules were prepared in accordance with the learning objectives.	◉	○	○	○	○	○
The length of the modules was appropriate.	◉	○	○	○	○	○
End of module questions were sufficient to evaluate my learning.	◉	○	○	○	○	○
I had technical problems while using the system.	◉	○	○	○	○	○
At the end of this training, I feel ready to work in the clinic.	◉	○	○	○	○	○
I can use what I learned in the clinic.	◉	○	○	○	○	○
I would like to receive a similar training organized in this way.	◉	○	○	○	○	○
I would like to share on the system with my friends who attend the course.	◉	○	○	○	○	○
I recommend this course to all physician candidates.	◉	○	○	○	○	○
I would have preferred a live lesson (with the lecturer's synchronous narration) in the course.	◉	○	○	○	○	○
I had internet access problem while completing this course.	◉	○	○	○	○	○

11 *

Evaluate your overall satisfaction with this course from 1 to 10.

1 I am not satisfied at all ------- 10 I am very satisfied

○ 1 ○ 2 ○ 3 ○ 4 ○ 5 ○ 6 ○ 7 ○ 8 ○ 9 ○ 10

12

What did you already know about the content of this course?

13

What are the main topics you have just learned with this course?

14

Which subjects would you like to have more detailed information about?

15

Want to add?

Close this window
